# Effectiveness of *Gamma Oryzanol* on prevention of surgical induced endometriosis development in rat model

**DOI:** 10.1038/s41598-022-06883-4

**Published:** 2022-02-18

**Authors:** Mohammad Yari Eisalou, Mohammad Reza Farahpour

**Affiliations:** 1 Department of Basic Sciences, Faculty of Veterinary Medicine, Urmia Branch, Islamic Azad University, Urmia, Iran; 2 Department of Clinical Sciences, Faculty of Veterinary Medicine, Urmia Branch, Islamic Azad University, 57159-44867 Urmia, Iran

**Keywords:** Biological techniques, Cell biology, Molecular biology

## Abstract

Infertility is believed to be triggered by endometriosis whose pathophysiology and the etiology is still unknown. Certain genes play pivotal roles in pathogenesis of endometriosis. Natural products and plants are used as important sources for production of new drugs. The current study assesses the effects of gamma-oryzanol (GO) in a rat model with surgically induced endometriosis. The experimental endometriosis was induced in 24 wistar rats, and the animals were subsequently subdivided into endometriosis-sole (endom group), 3000 and 6000 µg/kg GO (GO-3000 and GO-6000) and Vit C groups. The protein levels of estrogen receptor-alpha (ER-α), Steroidogenic factor 1 (SF1), Sirtuin 1 (SIRT1), heme oxygenase 1 (HO1), light chain 3 (LC3B) and Beclin1 (BECN1) were assessed. TUNEL staining was used for detecting the apoptosis rate. The results revealed that protein levels of SF1, HO1, and total LC3B significantly (*P* < 0.05) decreased in GO-6000-treated groups compared to endom group. Moreover, the protein level of BECN1 and SIRT-1 significantly (*P* < 0.05) increased in GO-6000-treated groups compared to endom group. GO treatment did not imply any significant difference (*P* > 0.05) concerning the protein levels of ER-α. The TUNEL staining results showed higher TUNEL-positive cells reactions in the rats treated with GO-6000 and Vit C. Thus, GO is involved in improving condition rats involved with endometriosis through modulation in the protein levels of some molecules and also induction of apoptosis.

## Introduction

Endometriosis is known as one of the most prevailing gynecologic diseases that is characterized by the presence of endometrial cells outside the uterine cavity^1,2^. It is diagnosed with common signs, such as dysmenorrhea, and infertility^3^. The pathophysiology and the etiology of the endometriosis are not still elucidated. According to recent evidences, the disease is an estrogen-dependent disorder whose symptoms might be cyclic parallel with menstrual cycle and significantly resolve after menopause, but not completely^4^. Studies have reported the increased expression of estrogen receptor-alpha (ER-α) during the proliferative phase in response to estrogen, and decreased the expression it during the window of implantation in response to progesterone in uterine tissue^5,6^. It has been also reported that the disappearance of ER-α during implantation is due to distribution of the expression pattern of the proteins controlling the endometrial receptivity^6^. Both ERα and ERβ are present in the endometrium, but ERα is seemingly a primary mediator of the estrogenic activity in this tissue^7^. Both receptors may be essential for the development and growth of endometriosis^6,7^.

Steroidogenic factor (SF)1 belongs to orphan nuclear receptors and is commonly evaluated in endometriosis. SF1 activates the full steroidogenic cascade of the genes such as aromatase, which converts cholesterol to estradiol in endometriotic cells and endometrium^8^. In vivo SF1 expression encourages the progression of enlarged endometrial glands and decreases estrogen and progesterone responsiveness in ectopic endometrial tissue^9^. SF1 is essential for activation of multiple steroidogenic genes for estrogen biosynthesis and its expression aberrantly is to be increased in endometriotic cells^8^. Sirtuin 1 (SIRT1) acts as a tumor promoter in endometrial cancer by targeting sterol regulatory element binding with protein 1 (SREBP1) and lipogenesis^10^. It also regulates inflammatory cytokines expression in endometriotic stromal cells^11^. Over-expression of SIRT1 possibly induces the pathogenesis of endometriosis in eutopic endometrium^2^. Heme oxygenase (HO) is characterized as a microsomal enzyme found in three isoforms (HO-1, -2, and -3), but HO-1 is inducible through some oxidant and non-oxidant stressors^12^. Allavena et al.^13^ reported increased the expression of HO-1 protein in patients suffering from ovarian endometriosis. Microtubule-associated protein light chain 3 (LC3) and Beclin1 (BECN1) are specific marker proteins of autophagy whose expressions are to be decreased in endometriosis patients^14^.

Natural products and plants are employed as important sources for producing new drugs^15,16^. Gamma-oryzanol (GO) is extensively found in the bran and germ of rice^17^ and has several biological properties, such as anti-oxidative, anti-inflammatory and wound healing activities^18–20^. The pathophysiology and the etiology of the endometriosis are still unknown, yet several studies have implied that antioxidants might be beneficial for these patients^16,21,22^, but the mechanism of action antioxidant in this disease is still unknown. GO might exhibit its effects via molecular mechanisms and/or via the induction of apoptosis. The genes of ER-α, SF1, SIRT1, HO-1, LC3B, and BECN1 are involved in the endometriosis. We hypothesized that GO shows its effects in improving the endometriosis via molecular mechanisms and induction of apoptosis. Therefore, the present work assesses the effects of GO in rat models with surgically induced endometriosis, by evaluating the western blotting expression of ER-α, SF1, SIRT1, HO-1, LC3B, BECN1. Moreover, the apoptosis rate was assessed with immunohistochemical staining method.

## Results

### Western blot analysis of ER-α and SF1

**Figure 1:**
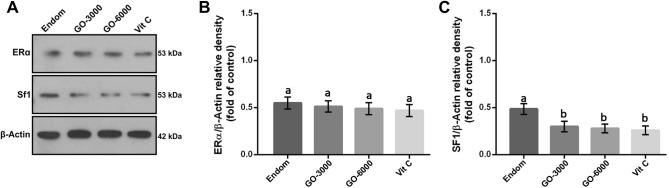
Effect of gamma-oryzanol on relative protein expressions assessed by Western blot. Representative Western blot bands of ER-α, SF1 and β-actin (**A**) and the relative protein expression of ER-α (**B**) and SF1 (**C**) in experimental groups. These proteins were normalized by β-actin and reported as relative change compared to endom-group. The results showed that the expression of SF1 decreased in the treated groups compared to the endom-group (*P* < 0.05). The superscripts (a–e) display differences among groups.

The results for Western blot analysis of ER-α and SF1 are represented in Fig. 1. Our findings did not show significant differences between groups (*P* > 0.05) concerning the levels of ER-α protein. Additionally, there were no significant differences between the endom group and the treatment groups regarding the levels of ER-α protein (*P* > 0.05). The findings also illustrated that the levels of SF1 protein significantly decreased in the treatment groups compared to the endom group (*P* < 0.05). Meanwhile, no significant differences were observed between Vit C group and the GO-3000 and GO-6000 groups (*P* > 0.05). The increasing levels of GO did not have any significant effects on the levels of ER-α and SF1 protein (*P* > 0.05).

### Western blot analysis of SIRT1 and HO

**Figure 2:**
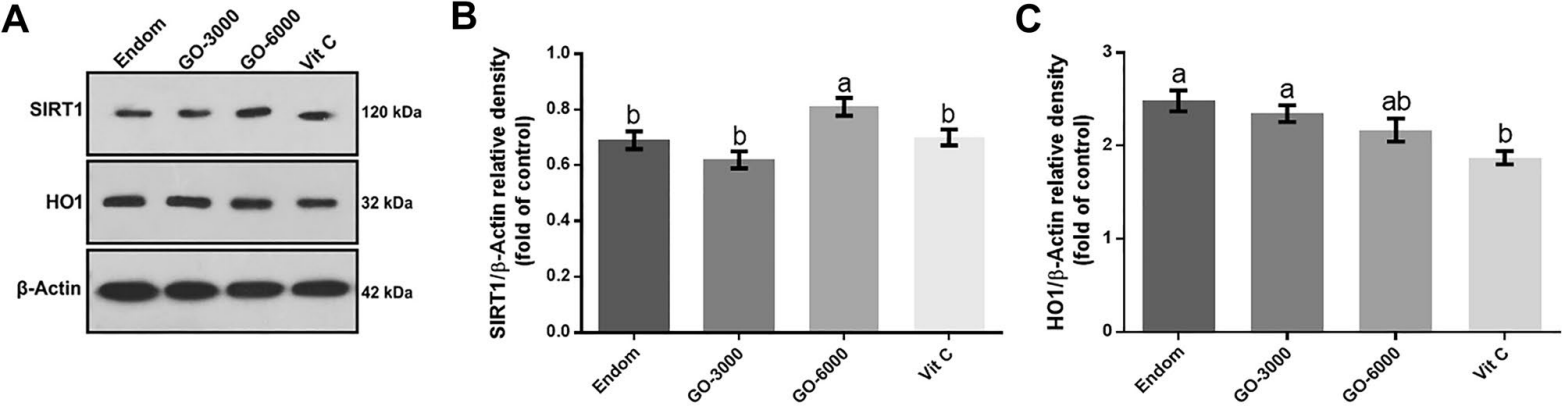
Effect of gamma-oryzanol on relative protein expressions assessed by Western blot. Representative Western blot bands of SIRT1, HO-1 and β-actin (**A**) and the relative protein expression of SIRT1 (**B**) and HO-1 (**C**) in experimental groups. These proteins were normalized by β-actin and reported as relative change compared to endom-group. The result showed the increased expression of SIRT1 in the rats treated with GO-6000 compared to the other groups (*P* < 0.05). The level of HO-1 protein significantly decreased in Vit C group compared to GO-3000 and endom-groups (*P* < *0.05*). The superscripts (a,b) display differences among groups.

The results regarding the levels of SIRT1 and HO-1 protein are presented in Fig. 2. The findings showed that the level of SIRT1 protein significantly increased in GO-6000 group compared to the other groups (*P* < 0.05). They did not exhibit any significant differences between the endom group and GO-3000 and Vit C group (*P* > 0.05). The results concerning the level of HO-1 protein indicated that the level of HO-1 was significantly lower in the rats treated with Vit C compared to the rats in the endom and GO-3000 groups (*P* < 0.05). No significant differences were observed neither between Vit C and GO-6000 group (*P* > 0.05), nor between the endom group and GO groups (*P* > 0.05).

### Western blot analysis of BECN1, LC3B

**Figure 3:**
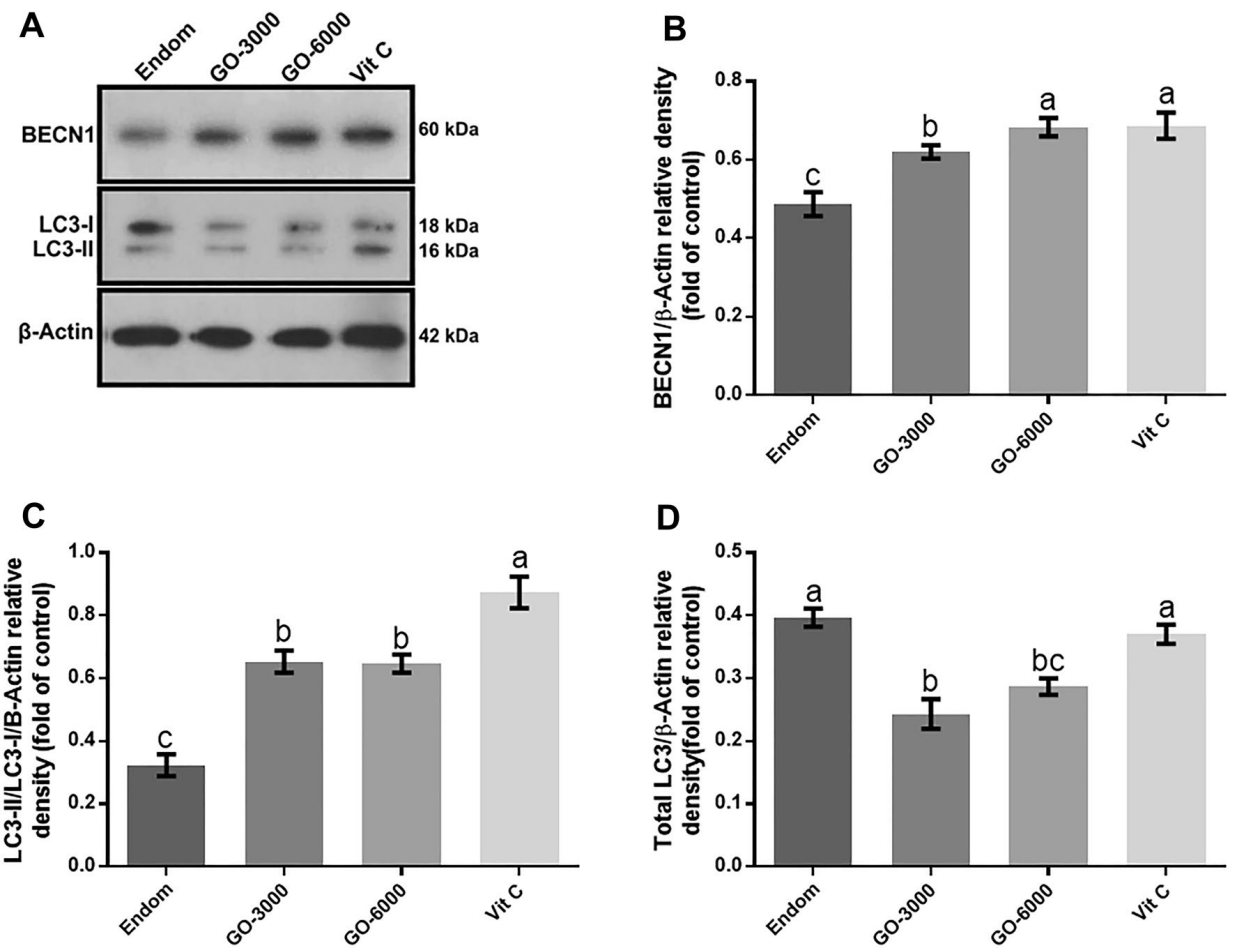
Effect of gamma-oryzanol on relative protein expressions assessed by Western blot. Representative Western blot bands of BECN1, LC3-II, Total LC3 and β-actin (**A**) and the relative protein expression of BECN1 (**B**), LC3 (**C**) and total LC3 (**D**) in experimental groups. These proteins were normalized by β-actin and values are expressed as relative change compared to endom-group. The results showed the increased level of BECN1 protein in the rats treated group compared to the endom group (*P* < 0.05). The level of LC3-II protein was significantly higher in Vit C group compared to the other groups (*P* < 0.05). The superscripts (a–c) display differences among groups.

The results about the levels of BECN1, LC3B-I and total LC3B-II protein are represented in Fig. 3. They showed the increased level of BECN1 protein in the treated groups compared to the endom group (*P* < 0.05). The level of BECN1 protein was reported to be significantly higher in Vit C and GO-6000 groups compared to GO-3000 group (*P* < 0.05). It means that with the increase in the levels of GO, the level of BECN1 protein significantly increases (*P* < 0.05). The rats treated with GO did not reveal any significant difference with the endom group for LC3-II (P > 0.05), yet the level of LC3-II protein was significantly higher in the rats treated with Vit C compared to the other groups (*P* < 0.05). Furthermore, the results did not show significant differences between the endom, GO-6000 and Vit C regarding the total LC3B (*P* > 0.05). No significant differences were seen in the rats treated with GO in connection with the total LC3 (*P* > 0.05).

### Number of TUNEL-positive cells

**Figure 4:**
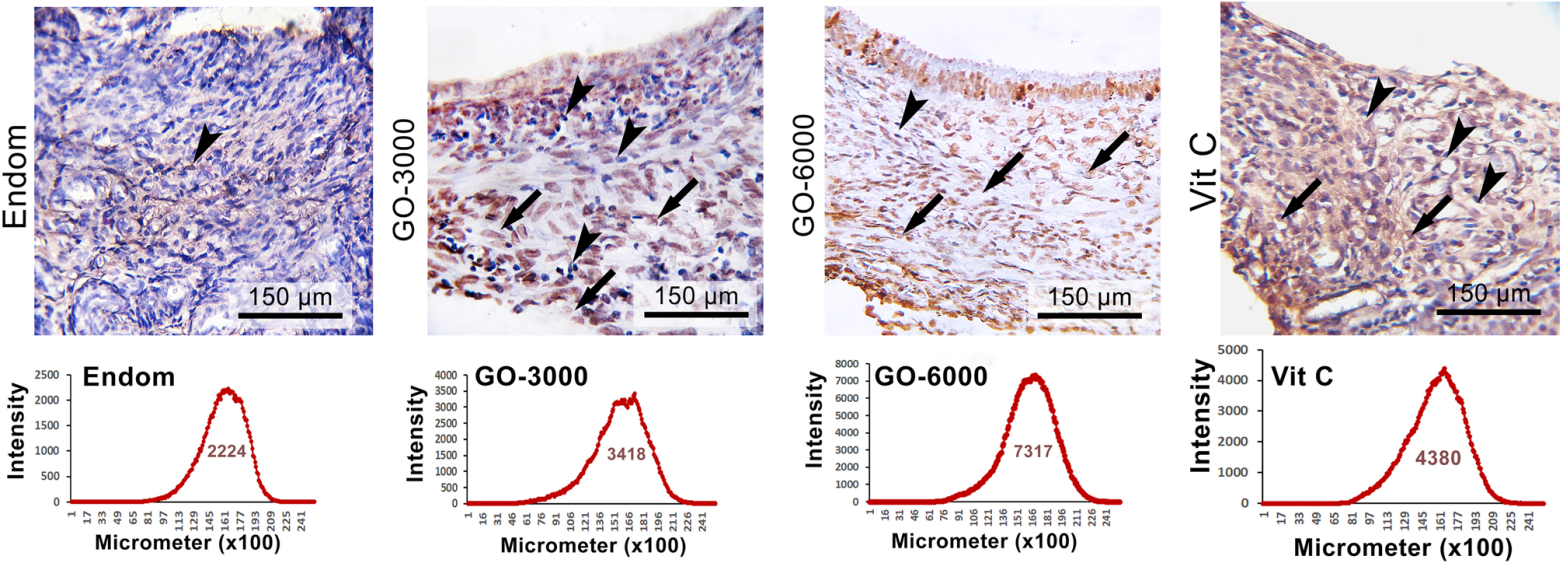
TUNEL staining of ectopic endometrial tissue. Of note, apoptotic cells increased in Vit C-treated GO-6000-treated groups compared to the endom and other experimental groups. As could be seen, the lowest and the highest TUNEL reactions belonged to the section from the endom-group and the section from Vit C and GO-6000, respectively.

The results for TUNEL-positive cells staining showed higher TUNEL-positive cells reactions in the rats treated with GO-6000 and Vit C compared to the other groups (Fig. 4). It means that GO-6000 and Vit C act as inducer apoptosis (Fig. 4).

## Discussion

The pathophysiology of the endometriosis is not known. Anti-estrogen agents^23,24^ and dopamine agonists^25^ are broadly used for the treatment of the endometriosis. Antioxidant agents are known to have beneficial effects for the treatment of endometriosis^4,16,21^. In the present study, we induced endometriosis in the rats and treated them with GO and Vit C as standard group. The protein levels of ER-α, SF1, SIRT1, HO-1, LC3 and BECN1 were assessed with western blotting. In the following, we will discuss protein levels of ER-α, SF1, SIRT1, HO-1, LC3 and BECN1.

The obtained results showed that the protein level of ER-α was not different between the groups (*P* > 0.05). It means that Vit C and GO in both levels did not significantly affect the protein level of ER-α. Both ERα and ERβ are present in the endometrium, but ERα is mostly involved in the endometriosis^7^. A review article implied that ERβ is mainly involved in this disorder^7^. Both ER-α and ERβ are proteins with high affinity for estradiol, which are encoded with different genes and are solely controlled in endometriosis compared to eutopic endometrium. To sum up, previous studies have shown that ER-α can have effects on endometriosis, but our findings implied that GO and Vit C could not affect the protein levels of ER-α. Unfortunately, no studies have been conducted to evaluate the effects of GO on the expression of ER-α. It was reported that rutin, as a natural agent, increased estradiol concentration in serum and mammary glands^26^. Other studies on other flavonoids showed that these increased E2 levels in mammary gland of pubertal female rats^27^. Liu et al.^28^ suggested that rutin alleviates the ischemia/reperfusion injury in ovariectomized rats through intervention in ER-mediated signaling pathways. However, GO and Vit C cannot act similarly to the mentioned flavonoids.

Herein, our findings indicated that the endom group increased the protein level of SF1, but Vit C and GO decreased its level. It means that GO and Vit C are of similar efficiencies for decreasing SF1. SF1 induces a steroidogenic cascade of genes, such as aromatase that converts cholesterol to estradiol^8^. In vivo SF1 expression promotes the progression of enlarged endometrial glands and decreased estrogen and progesterone responsiveness^9^. In the present study, GO and Vit C decreased the expression of SF1. With regards to the role of SF1 in the promotion of enlarged endometrial glands and conversion of cholesterol to estradiol, GO and Vit C decrease the expression of SF1, resulting in the decrease production of enlarged endometrial glands.

The expression of SIRT-1 was higher in GO-6000. The increase in the expression of SIRT1 increases the pathogenesis of endometriosis in eutopic endometrium^2^. SIRT1 deacetylates both histones and non-histone proteins, including p53^29^. Deacetylation action by SIRT1 enables it to control gene transcription and influence some cellular processes^30^. Vit C and GO-3000 did not have significant effects on the protein level of SIRT-1. The increased protein level of SIRT-1 in GO-6000 is unknown. The results showed that GO-6000 and Vit C act as inducer apoptosis and may decrease pathogenesis by inducing apoptosis as marked by TUNEL staining.

The expression of HO-1 decreased in Vit C group, but GO-3000 did not indicate such effect. The animals in GO-6000 treatment did not show any significant differences compared to those in Vit C group. The increased hemoglobin in the peritoneal cavity in this disorder may be a result of enhanced influx created by red blood cell degradation, due to increased retrograde menstrual reflux or from endometriotic lesions bleeding and/or faulted hemoglobin in activating system. On the other hand, cells mainly protect themselves from hemoglobin and heme toxicity by rapid expression of scavenger proteins such as HO-1^15,31^. We believe that Vit C decreases HO-1 levels by its antioxidant properties. GO-3000 could not have the similar effects as Vit C, which could be attributed to its antioxidant properties. GO-6000 has more antioxidant properties and show better effects. It also plays the roles by BECN1 gene, as will discussed.

The result revealed the level of BECN1 protein in the rats treated group increased compared to the endom group (*P* < 0.05). BECN1 family genes are known to have roles in apoptosis process^32^. The amount of BECN1 gene is increased in autophagy gene, which increases the production of autophagy vesicles and plays role in protecting cells against chromosome instability^33^. BECN1 plays an important role in induction of apoptosis in endometriosis cells. Apoptosis process eliminates endometriotic cells without the induction of inflammation. It was reported higher expression of anti-apoptotic factor and decreased expression of pro-apoptotic factors in eutopic endometrium compared to healthy women^34^. Thus, GO and vitamin C induce apoptosis by BECN1 and decrease endometriosis. The results for apoptosis was confirmed by TUNEL staining.

The level of LC3-II protein was significantly higher in Vit C group compared to other groups (*P* < 0.05), but the level of total LC3 protein decreased in GO-3000 group compared to endom and Vit C groups. LC3 is a specific marker that can directly show the level of autophagy^35^. The findings demonstrated that GO cannot act similarly to Vit C for the protein level of LC3.

In sum, the effects of gamma-oryzanol as a therapeutic agent to alleviate pathogenesis of endometriosis in rats were investigated by the protein level of ER-α, SF1, SIRT1, HO-1, LC3 and BECN1. Our results showed that GO is involved in decreasing the pathogenesis of induced endometriosis by influencing on BECN1 and SF1, and also inducing apoptosis in rats. This was a preliminary study and future studies are required to investigate the effects of GO on endometriosis. We suggest staining by immunofluorescent for investigation of makers in future studies.

## Materials and methods

### Materials

Gamma-oryzanol (Cat No: S0292 SIGMA) was prepared from, Sigma Chemical Co. (St. Louis, MO, USA). The antibodies included anti ER-α (sc-65718), anti SF1 (sc-398202), anti SIRT1 (sc-74465), anti HO-1 (sc-136960), anti BECN1 (sc-48341), anti LC3B (ab51520) and β-actin (sc-47778), which were prepared from Santa Cruz Biotechnology and Abcam (Cambridge, the United Kingdom). Other chemicals were prepared in form of commercial products of analytical grade.

### Animal model

Herein, 24 female Wistar albino rats, non-pregnant and nulligravid, were obtained from Pasture Institute (Amol-Iran). The rats had an age mean of 11 ± 1.2 weeks and weight of 180 ± 20 g. They were kept and studied in a standard environmental condition, with the temperature of 21 ± 3 °C and humidity of 60 ± 5%, and involved 12 h L/12 h D cycles. Standard pellet and water were freely provided for the animals. The animals were fed antibiotic free food (Javeneh Khorasan Company-Iran) and water ad libitum; they were housed and maintained in accordance with Iranian ethical guidelines for the use of animals. All the used protocols were in agreement with ARRIVE guidelines such as study design, sample size, randomization, outcome measures, data analysis, experimental procedures, reporting the results, etc., in this study were approved by the Committee on the Ethics of Animal Experiments of Veterinary Faculty and the Islamic Azad University Council on Animal Care, Urmia, Iran (IAUIAC permit number: FW2020-162424241), in compliance with the Guide for the Care and Use of Laboratory Animals published by the US National Institutes of Health (NIH publication no. 85-23, revised 1996). We declare that all methods were performed in accordance with the relevant guidelines and regulations.

### Induction of endometriosis

Endometriosis was induced as reported by previous studies^16,36^. Briefly, the rats were anesthetized following administration of ketamine (100 mg/kg) and 2% xylazine (40 mg/kg). Following laparotomy, a midline incision was created for exposing the uterus and intestine. In order to remove the right uterine horns of the rats, standard hysterectomy was used and horns were cut into 4 mm^2^ square pieces. Single 6-0 nylon and 3-0 silk sutures were respectively used for suturing the serosa layer and abdomen.

### Grouping and treatments

Following the induction of endometriosis, the rats were divided into four groups, including endometriosis-sole (endom group), endometriosis-induced rats treated with 3000 and 6000 µg/kg GO (GO-3000 and GO-6000) and endometriosis-induced rats treated with 1300 mg/kg vitamin C (Vit C). The rats in endometriosis-sole group received 0.5 mL/kg of saline-normal. Vitamin C was taken into consideration as a positive standard. The rats in the treatment groups received agents orally for 4 weeks. The rats were treated once/day and the treatment was started 2 weeks after start of surgery.

To select dose, we selected animals (n = 24) with similar condition, induced endometriosis and orally treated them with 1500 µg/kg, 3000 µg/kg, 6000 µg/kg and 9000 µg/kg for 4 weeks. We also monitored animals for mortality, behavioral and toxicity signs during 28 days. We observed the best responses in 3000 µg/kg, and 6000 µg/kg and did not observe any mortality, behavioral and toxicity signs. Thus, we selected 3000 µg/kg, and 6000 µg/kg as selected doses in the current study.

### Protein extraction and Western Blot

The total amount of protein prepared from endometriotic cells and immunoblotting**/**western blotting was conducted as described previously^16,37^. The cells were harvested utilizing 1% Trypsin–EDTA and pelleted. For the sonication of the cell lysates, we used a sonication buffer containing 20 mM Tris–Hcl, 0.5 mM EDTA, 100 mM DEDTC, 1% Tween, 1 mM phenylmethylsulfonyl fluoride, and protease inhibitor cocktail tablets: complete EDTA-free (1 tablet/50 mL) and PhosStop (1 tablet/10 mL). Bradford method was employed for assessing the protein concentration^38^. Western blot analyses were performed as described by other researchers^39^. All the analyses were performed in a specific amount of protein (50 μg) and the samples were loaded and resolved by 12% SDS-PAGE. To remove nonspecific bonds, the membranes were blocked in a 5% BSA, containing buffer, for 2 h at room temperature, and they were subsequently incubated overnight with the desired primary antibody at its respective dilution at 4 °C. The membranes were washed with wash buffer TBST (50 mM/L Tris–HCl, pH 7.6, 150 mM/L NaCl, 0.1%Tween 20) and incubated at room temperature for 2 h with appropriate HRP-conjugated secondary antibody (1:15/000 dilution). Results are expressed in relative fold change compared to control (vehicle 1 h). The uncut gel images are presented in Supplementary Data.

### Immunohistochemical study for analysis of apoptosis rate

TUNEL staining was employed for the evaluation of the apoptosis ratio as reported by Labat-Moleur et al.^40^. Hematoxylin was used for counting the sections and was then dehydrated with ascending alcohol. Cells were considered apoptotic provided that they were observed to be clear and dark brown.

### Statistical analysis

The results were reported as mean data (± SD). The statistical analysis was performed by One-way ANOVA and by help of SPSS software. A P value less than 0.05 was considered as significant.

### Ethical approval

All the used protocols were approved by the Committee on the Ethics of Animal Experiments of Veterinary Faculty and the Islamic Azad University Council on Animal Care, Urmia, Iran (IAUIAC permit number: IAUU, No. 11119), and also by the US National Institutes of Health (NIH publication no. 85-23, revised 1996).

## Supplementary Information

Supplementary Information.

## Abbreviations

GO: Gamma-oryzanol
Vit C: Vitmain C
ER-α: Estrogen receptor-alpha
SF1: Steroidogenic factor 1
SIRT1: Sirtuin 1
HO1: Heme oxygenase 1
LC3B: Light chain 3
BECN1: Beclin1
TUNEL: Terminal deoxynucleotidyl transferase enzyme mediated dUTP nick end labeling
