# Supplementary material for: Effectiveness of Gamma Oryzanol on prevention of surgical induced endometriosis development in rat model
Source: Sci Rep. 2022 Feb 18;12:2816. doi: 10.1038/s41598-022-06883-4 (PMC8857219; doi:10.1038/s41598-022-06883-4)
Supplement: Supplementary file 1 — Supplementary Information. [file 41598_2022_6883_MOESM1_ESM.pdf]

Supporting Information to

Effectiveness of *Gamma Oryzanol* on prevention of surgical induced endometriosis development in rat model

Mohammad Yari Eisalou [1] and Mohammad Reza Farahpour*[2]

[a] Department of Basic Sciences, Faculty of Veterinary Medicine, Urmia Branch, Islamic Azad University, Urmia, Iran.

[b] Department of Clinical Sciences, Faculty of Veterinary Medicine, Urmia Branch, Islamic Azad University, Urmia, Iran.

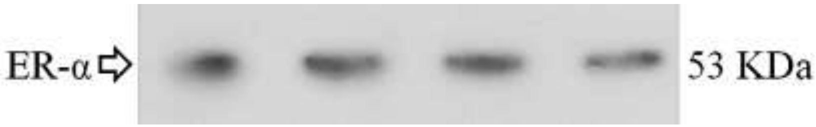

Figure 1: Full length gel of western blot for ER-α.

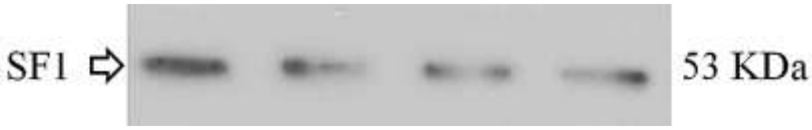

Figure 1: Full length gel of western blot for SF1.

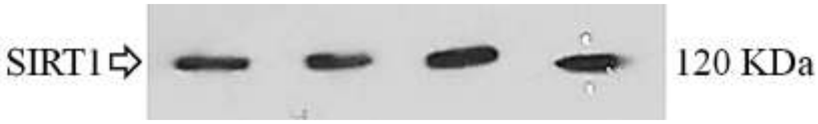

Figure 2: Full length gel of western blot for SIRT1.

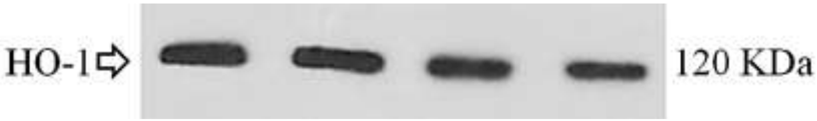

Figure 2: Full length gel of western blot for HO-1.

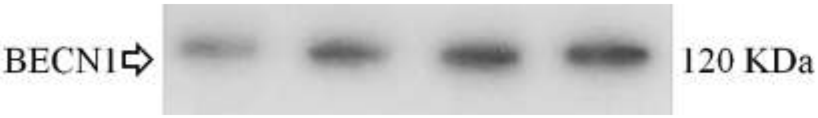

Figure 3: Full length gel of western blot for BECN1.

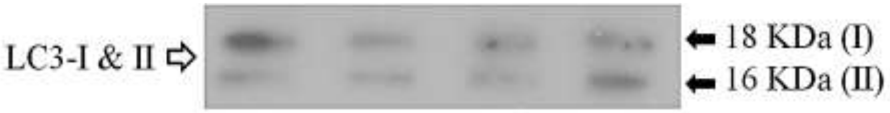

Figure 3: Full length gel of western blot for LC3-I & II.

Original images:

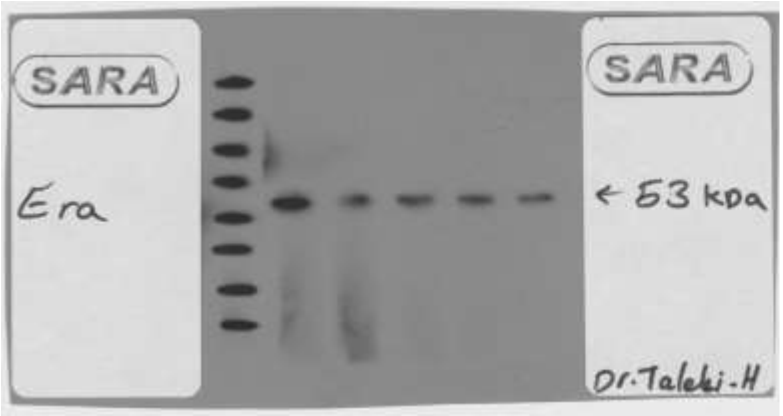

Figure 1: Full length gel of western blot for ER-α.

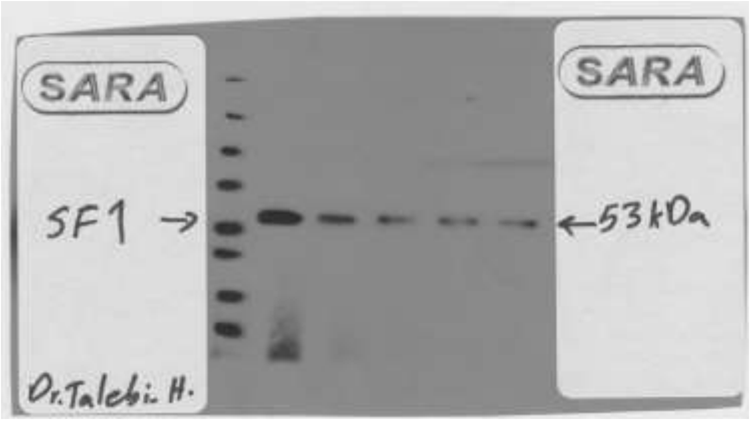

Figure 1: Full length gel of western blot for SF1.

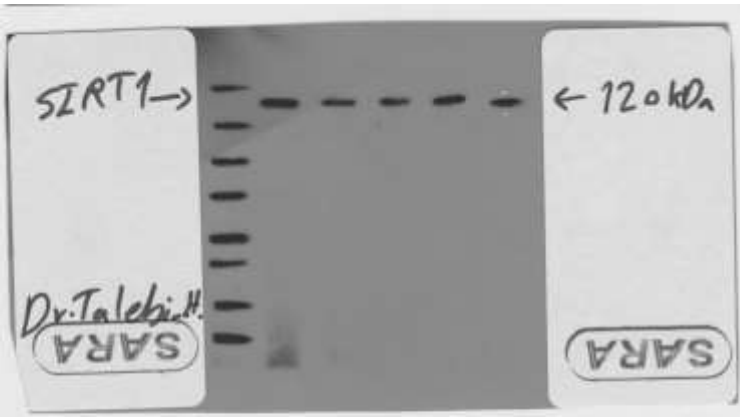

Figure 2: Full length gel of western blot for SIRT1.

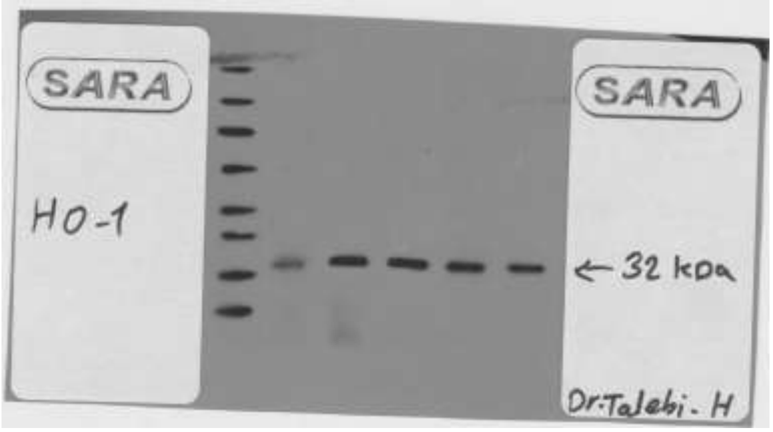

Figure 2: Full length gel of western blot for HO-1.

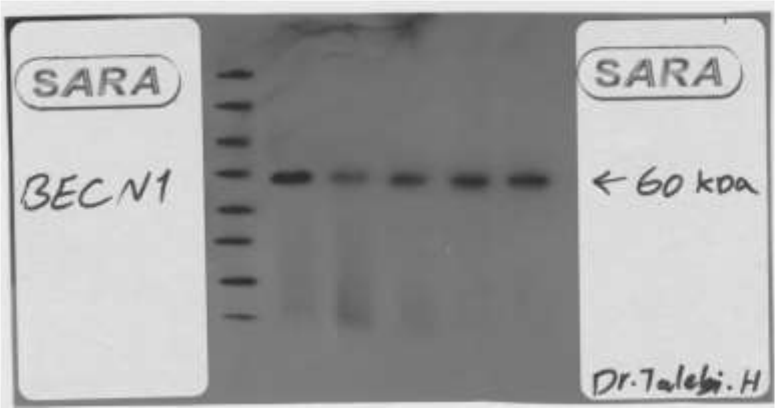

Figure 3: Full length gel of western blot for BECN1.

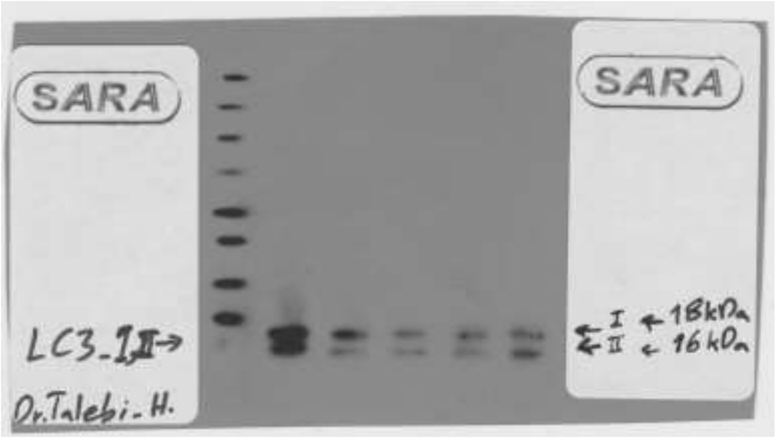

Figure 3: Full length gel of western blot for LC3-I & II.

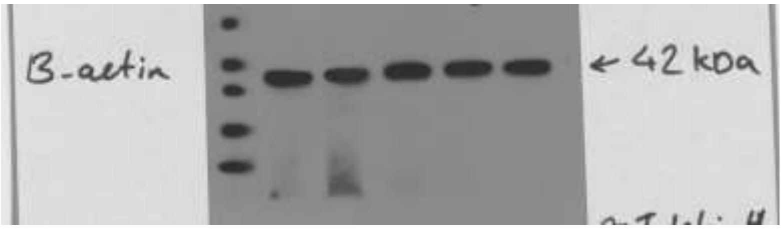

Figure : Full length gel of western blot for β-actin.
